# A Cross-Sectional Study of Obesity Effects on the Metabolomic Profile of a Leptin-Resistant Swine Model

**DOI:** 10.3390/metabo10030089

**Published:** 2020-03-05

**Authors:** M. Victoria Sanz-Fernandez, Laura Torres-Rovira, Jose L. Pesantez-Pacheco, Marta Vazquez-Gomez, Consolacion Garcia-Contreras, Susana Astiz, Antonio Gonzalez-Bulnes

**Affiliations:** 1 Subdirección General de Investigación y Tecnología-Instituto Nacional de Investigación y Tecnología Agraria y Alimentaria (SGIT-INIA), 28040 Madrid, Spain; mvsanzfernandez@gmail.com (M.V.S.-F.); mandymundi@hotmail.com (L.T.-R.); jose.pesantez@ucuenca.edu.ec (J.L.P.-P.); congarcon@gmail.com (C.G.-C.); astiz.susana@inia.es (S.A.); 2 School of Veterinary Medicine and Zootechnics, Faculty of Agricultural Sciences, University of Cuenca, 010220 Cuenca, Ecuador; 3 Faculty of Veterinary Medicine, Universidad Complutense de Madrid (UCM), 28040 Madrid, Spain; mvgomez@ucm.es

**Keywords:** Iberian pig, metabolic syndrome, metabolite profile, metabolomics, obesity

## Abstract

Identifying metabolite signatures associated with obesity and related diseases might represent a valuable preventive and therapeutic tool to predict subjects at risk, establish an accurate prognosis, and monitor treatment success. The current cross-sectional study is aimed to evaluate the metabolite profile of diet-induced obesity in a porcine model of leptin resistance. Six Iberian female pigs prone to develop obesity (OB) were ad libitum fed a fat-enriched diet (HFD) for 82 days. Five lean Iberian sows (CON) in a maintenance diet served as controls. At the end of the dietary treatments, all animals were sacrificed, and plasma, liver, and muscle samples were immediately collected for nuclear magnetic resonance analysis. In plasma, signals corresponding to betaine, glycerophosphocholine/phosphocholine, glycine, and glutamate were decreased; and the valine signal was increased in OB sows compared to controls. Similarly, the betaine signal was decreased in the liver. No differences were detected in muscle. The observed metabolite changes suggest alterations in branched chain amino-acid metabolism and the methionine-homocysteine cycle, which have been previously associated with obesity-related diseases and type 2 diabetes in human observational studies. The current study supports the utilization of the leptin resistant Iberian pig for further interventional research in the field.

## 1. Introduction

Obesity has currently reached pandemic proportions worldwide and, hence, there is an increasing interest in identifying biomarkers of metabolic risk factors. For instance, early indicators of insulin resistance (IR) might detect individuals at higher risk of type 2 diabetes (T2D), allowing for the implementation of preventive measures. Further, appropriate markers could improve diagnosis accuracy, prognosis capacity, and serve to monitor the efficacy of treatment interventions. To this end, metabolomic techniques have proven to be an invaluable tool for the identification of metabolic signatures linked to obesity and related illnesses [1].

Alterations in glycolytic and tricarboxylic acid cycle intermediates, branched chain amino acids (BCAA), betaine or long-chain fatty acids concentrations have been associated with metabolic diseases [1]. Nevertheless, current knowledge has been mainly generated from human cohort studies, which frequently prevents from establishing the underlying biological mechanisms responsible for such changes. Interventional studies with appropriate animal models would allow to determine cause-effect relationships within obesity metabolic fingerprints. The pig is rapidly emerging as a biomedical model due to its similarities with humans from a digestive, metabolic, and cardiovascular point of view [2]. The Iberian pig has proven particularly valuable in obesity studies, as it is naturally leptin resistant, with exacerbated appetite and fattening. Moreover, we have previously demonstrated that the obese Iberian pig develops metabolic syndrome, resembling the human phenotype [3]. 

Our study objective was to evaluate the metabolite profile in a model of diet-induced obesity. For this purpose, we analyzed the metabolome of different tissues, utilizing high-resolution magic angle spinning (HR-MAS) proton nuclear magnetic resonance (H+ NMR) spectroscopy.

## 2. Results

Obese sows had increased body weight (BW) and back fat depth, both at the beginning (29 kg and 1.0 cm difference, respectively; *P* < 0.01) and end (78 kg and 3.7 cm difference, respectively; *P* < 0.01) of the experiment, compared to control (CON) sows.

**Figure 1:**
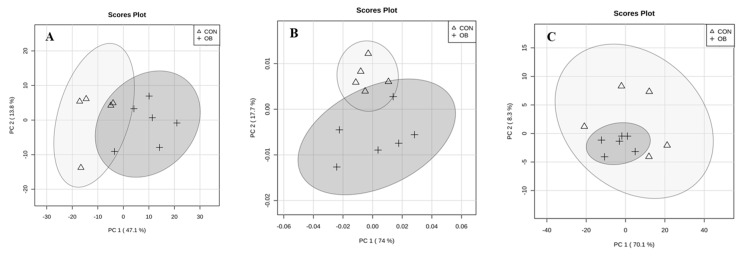
Principal component analysis of (**A**) plasma, (**B**) liver, and (**C**) muscle nuclear magnetic resonance results. Triangle: control sows (CON); cross: obese sows (OB).

In plasma, the principal components analysis (PCA) of the NOESY experiment indicated that seven components were necessary to explain 95% of the variance. Specifically, principal components 1, 2, and 3 (PC1, 2, and 3) accounted for 47.1%, 13.8%, and 11.3% of the variability, respectively. Treatment groups were well discriminated in PC1 and 2, being significantly different in PC1 (Figure 1A). No outliers were detected among samples. From the loading values of the variables associated with PC1 and PC2, we established the buckets (and their corresponding variables) with the highest contribution to treatment differences. The integrals for the signals corresponding to glycerophosphocholine or phosphocholine (GPCho/PCho, 3.23 ppm bucket), betaine (3.27 ppm bucket), and glycine (3.55 ppm bucket) were decreased (*P* < 0.01) in obesity (OB) sows. After suppressing broad signals from macromolecules (Carr-Purcell-Meibom-Gill, CPMG experiment), a decrease in valine (1.05 and 2.29 ppm buckets; *P* < 0.01) and an increase in glutamate (2.33 and 2.35 ppm buckets; *P* < 0.01) signals were detected in OB sows (Table 1). 

In the liver, three PCs were necessary to explain 95% of the variance for the NOESY experiment. Principal components 1, 2, and 3 accounted for 74.0%, 17.7%, and 4.2% of the variability, respectively. Treatment groups were well discriminated in PC1 and 2, being statistically different in PC2 (Figure 1B). No outliers were detected among samples. Only the integrals for the signals corresponding to betaine (3.28 ppm bucket) were significantly decreased (*P* < 0.01) in OB sows compared to controls. Additionally, in the CPMG experiment, the signal for the 2.24 ppm bucket was increased in OB sows (*P* < 0.01). This signal could not be identify with certainty, but might correspond to 4-aminoadipate (Table 1). In the muscle, the PCA showed no differences between treatments (Figure 1C) or buckets, after Bonferroni adjustment.

## 3. Discussion

The rapid development and popularization of high-throughput analysis has enabled the application of metabolomic techniques to the study of the pathophysiology of obesity-related diseases. The identification of biomarkers is essential in order to prevent, diagnose, and evaluate intervention efficacy, maximizing success options and cost-effectiveness. Herein, we utilized HR-MAS H+ NMR spectroscopy to characterize the metabolite profile of diet-induced obesity in an animal model.

In the current cross-sectional study, OB sows had an intrinsic propensity to gain weight, as demonstrated by differences in initial phenotypic traits. The biological reasons behind such differences are currently unknown, but were independent of genetic background, diet, and management. To further exacerbate this phenotype, obesity was induced in OB sows with a fat-enriched diet (HFD). Excessive weight gain and fattening capacity are characteristic features of the Iberian pig. The increased ability to store adipose tissue represents an adaptive mechanism to seasonal cycles, where fat accumulation during periods of feed abundance enables survival during times of scarcity. Similar adaptations apply to humans, due to the hunter-gatherers’ feast and famine lifestyle of prehistoric ancestors [4]. 

Increases in circulating BCAA (leucine, isoleucine, valine) have been consistently associated with obesity and T2D, which agrees with the current study, where valine was elevated in OB sows. Valine is increased in obese individuals compared to lean controls [5] and its concentration is directly associated with BW and size, the homeostatic model assessment (HOMA) and circulating triglycerides [6]. Further, valine levels are predictive of T2D, as non-diabetics with high plasma concentrations have higher odds of developing the disease [7]. Similarly, glutamate and the glutamate to glutamine ratio have been positively correlated to metabolic syndrome-related variables and obesity [6,8,9]. Intriguingly, plasma glutamate was decreased in OB sows, which does not have an apparent explanation, but might be due to intrinsic characteristics of pigs. 

Among the observed changes, betaine was the only metabolite that was decreased, both in the plasma and in the liver of OB sows. Betaine has been traditionally considered an osmolyte and a methyl donor; however, recent reports highlight its importance in the regulation of systemic bioenergetics and metabolism [10]. Similarly, to our observations, lower serum betaine levels in humans are associated with increased adiposity indices in a dose-dependent manner [11]. Further, circulating betaine is inversely correlated to insulin sensitivity [12], plasma insulin and triglycerides, and the HOMA [6]. Betaine levels were also predictive of T2D in a high-risk population (elevated body mass index (BMI), increased fasting glucose and glucose intolerance), where higher plasma concentrations were associated with a reduced incidence [9]. In addition, plasma betaine might represent a suitable tool to monitor preventive strategies, as it increased after lifestyle intervention, being associated to reduced diabetes incidence [9]. As expected from the benefits derived of a high plasma concentration, dietary betaine supplementation in human and animal models ameliorates the metabolic consequences of obesity, improving BW and composition, glucose homeostasis, and hepatic lipid accumulation [12,13,14].

Mirroring the betaine results, both GPCho/PCho and glycine were decreased in the OB sows’ plasma. Due to its structural similarities, discriminating between choline derivatives is technically difficult, which prevents us from specifically differentiating between the two compounds [15]. Regardless, GPCho and PCho are metabolically related to choline, which is inversely associated with BW and/or adiposity parameters [11,16]. Decreased GPCho itself is related to increased fasting glucose [6]. Likewise, glycine has been negatively associated with BMI, visceral adiposity, the HOMA, circulating triglycerides and blood pressure [6]. Further, low glycine concentrations are predictive of T2D [1]. 

Remarkably, the three metabolites (betaine, choline, and glycine) are involved in the methionine-homocysteine cycle, which might explain their synchronized changes. Systemic betaine is either derived from the diet or de novo synthesized from choline [17]. Betaine acts as a methyl donor in the conversion of homocysteine into methionine, yielding dimethylglycine, which is further metabolized to glycine [12]. Homocysteine is regenerated from methionine through the successive synthesis of S-adenosylmethionine (SAM) and S-adenosylhomocysteine (SAH), and back to homocysteine. The conversion of SAM to SAH can be catalyzed by several enzymes, involving glycine and choline derivatives [10,13,17]. Ultimately, lower betaine, choline, and/or glycine concentrations might prevent the methylation of homocysteine to methionine, resulting in the accumulation of the former. In agreement, decreased plasma betaine levels are inversely related to circulating homocysteine [18]. Increased homocysteine is considered a risk factor for cardiovascular disease independently of traditional factors like dyslipidemia, hypertension, and insulin resistance [19,20]. Thus, changes in plasma betaine, choline, and glycine in the OB sows might be indicative of alterations in homocysteine/methionine metabolism, which also appear to be affected in metabolically unhealthy subjects.

In the present study, the metabolite profiles of the two treatments were best discriminated in plasma. Since blood metabolite concentrations are the result of systemic metabolism, this might suggest that a whole-body approach is essential in order to fully understand the consequences of obesity. In summary, several obesity/IR markers previously identified in human observational studies were similarly altered in the diet-induced obese Iberian pig. These results support the utilization of the current model for interventional studies on the metabolic fingerprint associated with the susceptibility to, the prognosis of and the recovery from obesity and related diseases. 

## 4. Materials and Methods

### 4.1. Animals and Experimental Design

All procedures involving animals were approved by the Scientific Ethics Committee of the Instituto Nacional de Investigacion y Tecnologia Agraria y Alimentaria and performed according to the Spanish (RD1201/05) and European Union (Directive 86/609) regulations regarding the protection of experimental animals. Six Iberian sows prone to develop obesity (OB) were selected among INIA’s herd. These were the heaviest animals despite receiving the same management as the rest of the lot. Matching normal body weight (BW) controls (*n* = 6; CON) were chosen. Sows were 3 years old and were all sisters from the same father. Animals were housed, in group, by treatment. Prior to the initiation of the experiment, all animals were feed-restricted as per standard practice (in order to avoid excessive fattening) a grain-based diet calculated to meet their predicted maintenance requirements (2 kg/d), with 4.2% total fat and 1% saturated fat. From the initiation of the experiment, OB sows were *ad libitum* fed (~7 kg/d) a fat-enriched diet (HFD; 6.3% total fat and 1.9% saturated fat) for 82 days. Control animals remained in the maintenance diet throughout the experiment. A CON sow was culled from the study due to illness and its data was not included in the analysis. 

Body weight was determined on days 0, 42, and 82 of experiment. Simultaneously, back fat depth was measured ultrasonically (SonoSite S-Series, SonoSite Inc., Bothell, WA) at the level of the head of the last right rib. At the end of the experiment, sows were sacrificed using the captive bolt technique followed by exsanguination and all the animals were sampled at the same time for assuring consistency of the results obtained [21]. Heparinized blood samples were obtained and chilled until plasma was separated by centrifugation (1500× *g*, 10 min). Liver and muscle (*longissimus dorsi*) samples were immediately harvested and snap frozen. All samples were kept at −80 °C until further analysis.

### 4.2. Nuclear Magnetic Resonance Data Acquisition and 2D Experiments

Plasma and intact liver and muscle samples were examined using HR-MAS, in agreement with protocols previously reported [22,23]. Proton NMR spectroscopy was performed at 500.13 MHz and 4 °C to minimize metabolic changes using a Bruker AVIII500 spectrometer 11.7 T. 

Nuclear magnetic resonance data acquisition: Plasma, and intact liver and muscle tissue were examined using HR-MAS, operating at 4 °C to minimize tissue degradation. Proton NMR spectroscopy was performed at 500.13 MHz using a Bruker AVIII500 spectrometer 11.7 T. Samples were placed inside a 50 L zirconium oxide rotor with cylindrical insert and spun at a 5000 Hz to remove the effects of spinning side bands from the acquired spectra. Shimming and NMR preparation were performed at 4 °C to minimize metabolic changes. Under such conditions, no noticeable degradation was observed during acquisition. Standard solvent suppressed spectra were acquired into 32 k data points and averaged over 256 acquisitions. Total acquisition lasted 20 min using a sequence based on the first increment of the NOESY pulse sequence (relaxation delay-90°-t1-90°-tm-90°-acquire free induction decay (FID)), in which a secondary radio frequency irradiation field is applied at the water resonance frequency during the relaxation delay of 2 s and during the mixing period (tm = 150 ms), with t1 fixed at 3 s. A spectral width of 6009.62 Hz was used. Additionally, a Carr-Purcell-Meibom-Gill (CPMG) pulse sequence (90-(-180-)n acquisition) was used as a T2 filter to suppress broad signals from macromolecules, allowing for the identification of small metabolites. These experiments were performed under rotor-synchronized conditions by setting the delay equal to 1/spin rate (200 s). A total spin-echo time of 100 ms was used to attenuate signals of high molecular weight. Water suppression was achieved through irradiation of water signal during 2 s. The following parameters were used: 256 scans, a spectral width of 6009 Hz and 32 k data points. All spectra were processed using TOPSPIN software, version 3.5 (Bruker Rheinstetten, Germany). Prior to Fourier transformation, the FIDs were multiplied by an exponential weight function corresponding to a line broadening of 0.3 Hz. Spectra were phased, baseline-corrected and referenced to the sodium (3-trimethylsilyl)-2,2,3,3-tetradeuteriopropionate singlet at δ 0 ppm.

The 2D experiment: for the components assignments, a number of 1H, 1H 2D experiments were performed. Gradient selected COSY90 was acquired under the following conditions: water presaturation during relaxation delay, spectral width of 6009 Hz in both dimensions, 2 k data points in f2, and 384 increments in f1. An unshifted sinusoidal window function was applied in both dimensions and zero filling in f1 dimension. Moreover, 1H, 1H TOCSY was registered in TPPI phase sensitive mode, with water presaturation during 1 s relaxation delay, a spectral width of 6002 Hz in both dimensions, a 70 ms mixing time, 2 k data points in f2, and 384 increments in f1. Zero filling in f1 and unshifted squared sinusoidal window function in both dimensions were applied before Fourier transformation. Furthermore, 1H, 13C 2D experiments were performed for the components assignments. HMQC experiments were registered with the following parameters: 70 μs for GARP 13C decoupling, 6009 Hz and 22 kHz spectral widths in the 1H and 13C dimensions, respectively; 2 k data points in f2, and 256 increments in f1. Zero filling in f1 and unshifted squared sinusoidal window function in both dimensions were applied before Fourier transformation.

### 4.3. Statistical Analysis

Production data was statistically analyzed using the MIXED procedure of SAS 9.4 (SAS Institute Inc., NC, USA). Multivariate statistical algorithms were used to classify HR-MAS 1H NMR spectra. For pattern recognition analysis, 1H NMR spectra were reduced using AMIX 3.6.8 (Bruker Rheinstetten, Germany) by subdivision into integral regions of 0.02–0.04 ppm excluding the water region. Individual regions were normalized to the total sum of integral region. Principal components analysis (PCA) was applied to the data without scaling. Loading plots from the PCA were used to identify the peaks responsible for significant differences. Peaks were associated with specific metabolites based upon existing literature. Comparisons were carried out using a Student’s t-test and Bonferroni adjustment. Data are reported as least square means and standard error of the mean, and considered significant if *P* < 0.05.

## Figures and Tables

**Table 1:** Effects of diet-induced obesity on changes in the metabolomic profile of a swine model.

Intensity, A.U.	Control		Obese		Trt ^1^
	LSMEAN	SEM	LSMEAN	SEM	*P*-Value
Plasma					
Betaine (3.27 ppm)	0.00936	0.00026	0.00565	0.00024	<0.0001
GPCho ^2^ or PCho ^3^ (3.23 ppm)	0.00921	0.00015	0.00797	0.00014	0.0002
Glycine (3.55 ppm)	0.00712	0.00016	0.00535	0.00015	<0.0001
Glutamate (2.35 ppm)	0.00457	0.00023	0.00218	0.00021	<0.0001
Valine (2.29 ppm)	0.00144	0.00003	0.00176	0.00003	<0.0001
Liver					
Betaine (3.28 ppm)	0.02668	0.00082	0.01741	0.00075	<0.0001
4-aminoadipate (2.24 ppm)	0.00054	0.00004	0.00102	0.00004	<0.0001

^1^ Treatment; ^2^ Glycerophosphocholine; ^3^ Phosphocholine.
